# Esophageal Lichen Planus: Highlighting Diagnostic Challenges and Therapeutic Limitations

**DOI:** 10.14309/crj.0000000000002202

**Published:** 2026-06-19

**Authors:** Arman Bahmani, Thomas Kemmerly, Brian E. Yang

**Affiliations:** 1 Department of Internal Medicine, Loma Linda University Medical Center, Murrieta, CA; 2 Department of Gastroenterology, Loma Linda University Medical Center, Murrieta, CA

**Keywords:** esophagitis, lichen planus, esophagus, dysphagia

## Abstract

Esophageal lichen planus (ELP) is a rare inflammatory disorder frequently misdiagnosed due to its nonspecific symptoms and overlapping features with other esophageal conditions. We present 3 cases of ELP characterized by delayed diagnosis and variable response to conventional therapies. All patients exhibited dysphagia and odynophagia with limited response to proton pump inhibitors, and endoscopic findings including mucosal sloughing, ulcerations, and strictures. Histopathologic findings were nondiagnostic but demonstrated features consistent with a lichenoid pattern of injury. While all patients trialed swallowed topical corticosteroids, 2 demonstrated minimal clinical response, and 1 required serial esophageal dilations and enteral nutritional support. These cases highlight the diagnostic and therapeutic challenges of ELP, emphasizing the need for increased awareness and development of standard treatment protocols.

## INTRODUCTION

Esophageal lichen planus (ELP) is an under-recognized condition primarily affecting middle-aged women and is frequently misdiagnosed as other forms of esophagitis. Dysphagia is the principal presenting symptom, and diagnosis is often delayed due to variability in clinical, endoscopic, and histopathologic findings. Endoscopic findings include mucosal denudation, tearing, hyperkeratosis, and trachealization, while histologic findings may demonstrate mucosal detachment, T-lymphocytic infiltrations, epithelial apoptosis, dyskeratosis, and hyperkeratosis.^1–3^ Despite increasing recognition, standardized treatment strategies remain lacking. Topical corticosteroids are generally first-line therapy, with endoscopic dilation considered for symptomatic strictures. This case series aimed to highlight the diagnostic and treatment challenges, overlap with other esophageal conditions, and enhance ELP recognition.

## CASE REPORT

### Case 1

**Figure 1.**
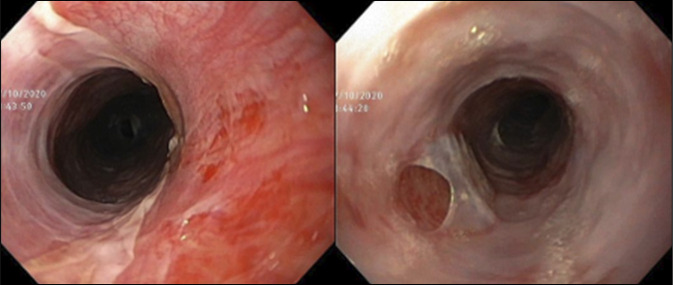
Upper endoscopy demonstrating patchy desquamation of the esophageal mucosa.

A 70-year-old woman with genital lichen planus (LP), pemphigus, and Hashimoto's thyroiditis presented with 1 month of dysphagia and chest discomfort, palpitations with swallowing, and intermittent oropharyngeal blisters. Physical examination revealed whitish discoloration along the buccal mucosa without oropharyngeal lesions. Esophagogastroduodenoscopy (EGD) revealed patchy mucosal desquamation throughout the esophagus, initially concerning for esophagitis dissecans (Figure 1). Biopsies revealed esophagitis without fungal organisms, dysplasia, or malignancy. She was treated with a proton pump inhibitor (PPI) and swallowed budesonide with minimal improvement and developed progressive dysphagia with an 18 kg weight loss. Repeat endoscopy showed mid and distal strictures impassable by a 10 mm endoscope. Evaluation at multiple tertiary centers demonstrated largely nondiagnostic biopsies, showing reactive squamous mucosa without eosinophilia or dysplasia. One biopsy was interpreted as lymphocytic esophagitis with comment that findings could represent lichen planus but lacked a fully developed lichenoid infiltrate, although most biopsies did not support lymphocytic esophagitis. A working diagnosis of ELP was supported based on clinical presentation, endoscopic findings, and history of lichen planus despite nondiagnostic histopathology. Her course was marked by progressive stricturing requiring serial dilations and eventual gastrostomy tube placement for nutritional support. Initial dilation was to 12 Fr, followed by staged dilations every 3 weeks, progressing to >16 mm with a target of 20 mm. She demonstrated clinical improvement but remains dependent on blenderized foods. Escalation to systemic immunosuppression, including mycophenolate mofetil, was offered but declined.

### Case 2

**Figure 2.**
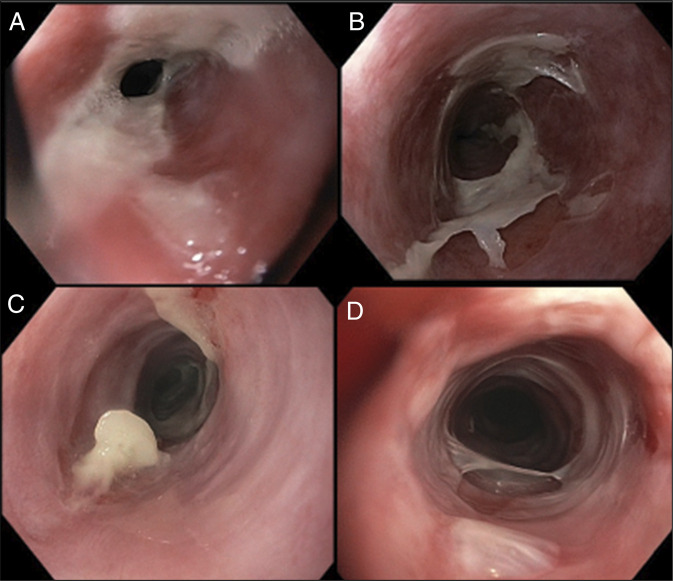
Endoscopic images showing proximal esophageal stricture with inflammatory mucosal changes (A), extensive sheets of sloughing mucosa, with areas of denudation (B), and repeat endoscopy with diffuse desquamation with friability (C) and patchy ulcerations (D).

A 51-year-old woman with hypertension and gastroesophageal reflux disease presented with 1 year of dysphagia to solids and liquids, associated with odynophagia, regurgitation, and 9 kg weight loss. Physical examination was unremarkable, and high-dose PPI trials provided minimal relief. Barium swallow showed delayed transit without structural abnormalities. EGD revealed proximal esophageal stricture and mucosal friability (Figure 2). Four biopsies were obtained, 2 from the proximal and 2 from the distal esophagus, demonstrating reflux esophagitis without infection, dysplasia, or malignancy. Symptoms persisted despite continued PPI therapy. Follow-up endoscopy showed diffuse desquamation, friability, and patchy ulcerations of the esophageal mucosa, concerning for ELP (Figure 2). Biopsies revealed spongiotic esophagitis with focal interface activity, epithelial clefting, and dyskeratotic cells in the proximal esophagus, and spongiotic dermatitis with granulation tissue in the distal esophagus. She was treated with swallowed budesonide without noticeable relief and referred for subspecialty evaluation.

### Case 3

**Figure 3.**
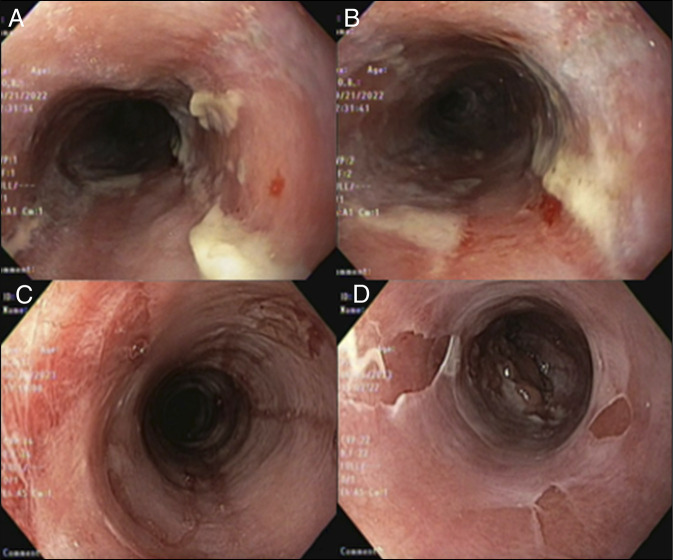
Upper endoscopy with Los Angeles grade C reflux esophagitis extending up to 6cm from the gastroesophageal junction (A) with several linear ulcerations (B). Repeat endoscopy showing subtle rings in the cervical esophagus, linear erosions from the distal to mid-esophagus (C), and friable, sloughing mucosa (D).

A 65-year-old woman presented with worsening reflux, ageusia, and intermittent dysphagia. Initial EGD revealed Grade C distal esophagitis with linear ulcerations extending 6 cm proximally from the gastroesophageal junction (Figure 3). Plans for empiric dilation were deferred due to active esophagitis. Biopsies from the proximal and distal esophagus showed severe erosive esophagitis without infection, dysplasia, or malignancy. Despite prolonged PPI therapy, symptoms persisted. Follow-up endoscopy demonstrated subtle proximal rings, linear erosions from the mid to distal esophagus, and friable mucosa suspicious for bullous esophageal disease or eosinophilic esophagitis (Figure 3). Plans for dilation were deferred. Proximal biopsies demonstrated squamous mucosa with inflamed granulation tissue without eosinophils, while distal biopsies revealed intraepithelial lymphocytosis, dyskeratotic keratinocytes, and acanthosis. Limited lamina propria precluded full assessment of a lichenoid inflammatory pattern. These findings were interpreted as a lichenoid pattern of injury but were not specific. However, given the persistent symptoms, proximal-predominant involvement, lack of response to PPI therapy, and evolving endoscopic findings, ELP was suspected. The patient was initiated on oral prednisone (40 mg daily) with a taper over several weeks before transitioning to swallowed budesonide, resulting in clinically meaningful improvement. Systemic immunosuppressive therapy was deferred.

## DISCUSSION

This case series highlights the diagnostic and therapeutic challenges associated with ELP. ELP is frequently misdiagnosed as more common esophageal disorders, including reflux esophagitis, pill-induced esophagitis, esophagitis dissecans superficialis, lymphocytic esophagitis, eosinophilic esophagitis, and autoimmune bullous disorders, resulting in delayed diagnosis.^1,4–7^ While dysphagia is the most common presenting symptom, variability in endoscopic and histopathologic findings contributes to diagnostic uncertainty. ELP may be asymptomatic despite advanced endoscopic disease or present initially with strictures, and although it can be the first manifestation of disease, isolated esophageal involvement is rare.^2,3,5^ Nonclassical feature, including isolated dysphagia, proximal strictures, or subtle endoscopic findings, particularly in the absence of extra-esophageal disease, further complicate recognition. Therefore, concomitant oral, genital, or cutaneous lichen planus should be actively considered.^1–6^

In our series, delayed diagnosis was due to misinterpretation of symptoms and lack of response to conventional treatments. Dysphagia persisted despite acid suppression therapy, with only 1 patient undergoing serial dilations and demonstrating clinical improvement.

The clinical presentations were marked by relative sparing of the gastroesophageal junction and lack of response to PPIs. Notably, all patients demonstrated mucosal sloughing and denudation, findings that overlap with esophagitis dissecans and are well described in ELP, further contributing to diagnostic ambiguity.^1,8^ Diagnosis therefore required integration of clinical evaluation, endoscopy, and histopathology. Endoscopic findings included webs, stenosis, friable and thickened mucosa with sloughing, superficial ulcerations, erosion, and subtle ring-like appearance.

Histopathologic findings were frequently nondiagnostic. Biopsies demonstrated reactive squamous mucosa, lymphocytic esophagitis, and features of a lichenoid pattern of injury, including intraepithelial lymphocytosis and dyskeratotic keratinocytes, without definitive confirmation. This overlap highlights the limitations of biopsy in ELP, particularly in early or patchy disease. Despite nondiagnostic histology, clinical suspicion remained high based on presentation, endoscopic findings, and lack of response to PPI therapy. Repeated nondiagnostic biopsies across multiple institutions further underscore the limitations of histopathology in ELP and the importance of clinical suspicion.

Management remains challenging due to the lack of standardized guidelines.^1,2,9^ Two of the 3 patients demonstrated limited response to topical steroids, and while historical approaches have favored topical or systemic steroids as first-line therapy, there is no consensus on treatment protocols.^2,10^ Larger cohort studies, including series of up to 132 patients, have demonstrated variable treatment responses in ELP, with topical corticosteroids showing inconsistent efficacy and a subset of patients requiring escalation to systemic immunosuppressive therapy.^3,6^ For severe or refractory cases, immunosuppressive agents such as rituximab, tacrolimus, cyclosporine, azathioprine, and mycophenolate mofetil have been reported, although evidence remains limited.^1,2^

The clinical implications are significant as delayed diagnosis increases the risk of progressive stenosis, nutritional compromise, and potential malignant transformation, as ELP is a recognized precursor to squamous cell carcinoma.^1,6,8^ These findings underscore the need for heightened clinical suspicion, particularly when biopsies are inconclusive and standard therapies fail.^2,5,9^ Further research is necessary to establish evidence-based protocols for managing ELP and improving patient outcomes.

## DISCLOSURES

Author contributions: A. Bahmani is the article guarantor, drafted the initial manuscript and performed the literature review. T. Kemmerly assisted in the retrieval of endoscopy images, fact-checked, critically reviewed, and edited the manuscript. B. Yang conceptualized the idea of this case series and provided final manuscript revisions.

Financial disclosure: None to report.

Informed consent was obtained for this case series.

## ABBREVIATIONS:

EGD: Esophagogastroduodenoscopy
ELP: Esophageal lichen planus
GEJ: Gastroesophageal Junction
LA: Los Angeles
LP: Lichen Planus
PPI: Proton Pump Inhibitor
